# Defining community‐led monitoring and its role in programme‐embedded learning: lessons from the Citizen Science Project in Malawi and South Africa

**DOI:** 10.1002/jia2.26277

**Published:** 2024-07-10

**Authors:** Krista J. Lauer, Melikhaya Soboyisi, Carol Ameera Kassam, Dennis Mseu, Gemma Oberth, Solange L. Baptiste

**Affiliations:** ^1^ International Treatment Preparedness Coalition ‐ Global (ITPC) Johannesburg South Africa; ^2^ The Networking HIV and AIDS Community of Southern Africa (NACOSA) Cape Town South Africa; ^3^ Independent Consultant Lilongwe Malawi; ^4^ Malawi Network of Religious Leaders Living with or Personally Affected by HIV and AIDS (MANERELA+) Lilongwe Malawi; ^5^ Center for Social Science Research University of Cape Town Cape Town South Africa; ^6^ International Treatment Preparedness Coalition – Global (ITPC) Johannesburg South Africa

**Keywords:** community‐led monitoring, COVID‐19, HIV, key populations, participatory action research, Programme Science

## Abstract

**Introduction:**

Programme Science (PS) and community‐led monitoring (CLM) intersect in unexpected and promising ways. This commentary examines a CLM initiative in Malawi and South Africa to highlight the crucial role of CLM in bolstering the PS framework. By leveraging data sources often overlooked by conventional research and evaluation approaches, CLM emerges as a pivotal element in enhancing programme effectiveness. This paper delineates the fundamental principles of CLM, presents programme outcomes derived from CLM methodologies and contextualizes these findings within the broader framework of PS.

**Discussion:**

The Citizen Science Project implements CLM continuously at 33 health facilities: 14 in Malawi (eight in Kasungu District and six in Dedza District), and 19 in South Africa (all in the West Rand District), representing a total catchment area of 989,848 people. Monitoring indicators are developed in an iterative process with community groups. The indicators are unique to each country, but both focus on the uptake of health services (quantitative) and barriers to access (qualitative). Monthly clinic records surveys capture 34 indicators in Malawi and 20 in South Africa and are supplemented by qualitative interviews with care recipients and healthcare workers. Qualitative interviews provide additional granularity and help confirm and explain the more macro trends in service coverage as described in quantitative data. The resulting data analysis reveals key themes that help stakeholders and decision‐makers to solve problems collaboratively. Noteworthy outcomes include a substantial increase in multi‐month dispensing of antiretroviral therapy (ART) during COVID‐19 (from 6% to 31%) with a subsequent recovery surpassing of HIV service benchmarks in Malawi post‐pandemic.

**Conclusions:**

While quantifying direct impact remains challenging due to the project's design, CLM proves to be a robust methodology that generates credible data and produces impactful outcomes. Its potential extends beyond the health sector, empowering community leadership and fostering interventions aligned with community needs. As CLM continues to evolve, its integration into PS promises to improve relevance, quality and impact across diverse disciplines.

## 1 INTRODUCTION

Community‐led monitoring (CLM) has the potential to strengthen programme‐embedded learning by centring the lived experiences of recipients of care, empowering them to generate and analyse their own data, and using the findings to advocate for the co‐creation and implementation of solutions.

Community‐led organizations extoll CLM as an effective mechanism for organized and systematic advocacy for the health and rights of key populations, addressing root causes of low service uptake and poor health outcomes [1, 2, 3]. Governments also emphasize the value‐add of CLM data for their own quality improvement efforts and comparison of facility‐level performance [4, 5]. Major funding partners either require [6] or recommend [7] its inclusion in their grants. Yet, CLM remains underutilized as a mechanism to improve access to, and quality of, health services [8, 9].

Programme Science (PS) is an iterative, multi‐phase research and programme framework where programmes drive the scientific inquiry, and both programme and science are aligned towards a collective goal of improving population health [10]. CLM is an accountability mechanism led and implemented by recipients of care that utilizes a structured platform and rigorously trained peer monitors to consistently gather and assess qualitative and quantitative data on service delivery, and to implement swift feedback loops with programme managers and health policymakers on areas requiring improvements [11]. If remedial actions fail to meet community expectations, advocates utilize CLM data to escalate their advocacy strategies and hold duty‐bearers accountable, including through public campaigns.

While CLM shares many principles in common with Participatory Action Research—both approaches focus on research tied to action; pay careful attention to power relationships; and situate data in their lived contexts [12]—CLM differs primarily in that it (a) mandates routine, recurring data collection that allows for the identification of trends and themes in the data, and also (b) leverages decision‐making structures in which community advocates can present data on programme gaps, facilitate the co‐creation of solutions and hold duty‐bearers accountable for change [13]. Although nascent, it is a rigorous methodology that generates credible data, achieves impactful outcomes and has the potential to elevate expert community leadership well beyond HIV and the health sector [5, 9, 14–16].

CLM has substantial potential to strengthen the PS framework by providing distinctive insights derived from community data, which may not be accessible through conventional research and evaluation approaches [2, 5]. CLM goes beyond quantitative analysis and reveals nuanced insights about the root causes of health systems gaps, bringing forth multifaceted, targeted solutions that are necessary to improve complex systems [17, 18]. CLM has led to demonstrable improvements in programme outcomes specifically because CLM data are used by communities in real time to hold duty‐bearers accountable for addressing identified gaps [3, 9, 15, 16]. The design of CLM upends the traditional public health hierarchy between passive “subjects” and powerful “investigators/researchers/experts,” positioning recipients of care as leaders who ensure that the issues being monitored and addressed are directly relevant to their lived experiences.

By examining a CLM programme in Malawi and South Africa, this paper defines the unifying principles underpinning CLM, describes a CLM implementation model, demonstrates CLM‐derived programme results, and discusses CLM's limitations and potential added value to PS.

### 1.1 Principles of the CLM methodology

CLM is a developing discipline with diverse implementation approaches. However, a white paper published by three civil society consortia in global health outlines shared CLM principles [19]. These principles prioritize community leadership over mere community involvement. Communities lead in identifying pressing issues, defining indicators, managing data, conducting analysis (recommended in partnership with an advisory group, academic institution or other data expertise) and advocating for remedial action on identified gaps. If issues persist, communities engage in advocacy to hold duty‐bearers accountable.

### 1.2 International Treatment Preparedness Coalition's CLM Implementation Model

This paper examines the specific implementation model pioneered by the International Treatment Preparedness Coalition (ITPC), comprised of four steps in a CLM cycle: Education, Evidence, Engagement and Advocacy [13].

### 1.3 Case Study: The Citizen Science CLM Programme in Malawi and South Africa

The Citizen Science project in Malawi and South Africa monitors the impact of COVID‐19 on HIV and tuberculosis (TB) services, with particular attention to prevention. Since October 2020, Citizen Science CLM has been taking place at 33 health facilities: 14 in Malawi (eight in Kasungu and six in Dedza), and 19 in South Africa (all in the West Rand). Together, they serve a catchment area of 989,848 people. The implementing partners are MANERELA+ (Malawi Network of Religious Leaders Living with or Personally Affected by HIV and AIDS, based in Malawi) and NACOSA (The Networking HIV and AIDS Community of Southern Africa, based in South Africa), in partnership with grassroots community groups Access Chapter 2 and Rotanganedza Community Care (South Africa).

### 1.4 Step 1: Education

ITPC briefs all parties on the methodology, process and purpose of CLM to establish a common understanding among both community and government partners, to mitigate scepticism and build trust. Community implementers and governments sign a Memorandum of Understanding that approves the monitoring effort and specifies monitoring sites. The scope of the briefings for government partners in Malawi comprises the Ministry of Health through the District Health office, and in South Africa, the Gauteng Department of Health, Chief Directors from the provincial Health District Directorate and Health Advisory Service Teams, the Clinical Forensic Services and Chief Directors, Directors, and Deputy Directors from various districts. The trust built between governments and community implementers over multi‐year implementation opens doors. A data‐sharing agreement in West Rand grants community implementers access to the government health information system, District Health Information System (DHIS2).

Communities receive education sessions about the science of HIV and global norms for prevention, treatment and care, followed by focus group discussions (FGDs) to determine the gaps between (a) the health services individuals should expect, and (b) their lived experiences.

### 1.5 Step 2: Evidence

Key issues emerge from FGDs and shape monitoring priorities, leading to context‐specific indicators unique to each CLM project in each country. Community implementers share the indicators with all relevant stakeholders, including health facility managers, municipalities and health departments to foster awareness of the areas under examination, to facilitate necessary approvals for data access and to minimize surprise when community findings are presented. Table 1 provides a sample of CLM indicators.

**Table 1:** Indicative quantitative and qualitative indicators for HIV‐focused CLM programmes and related advocacy [13]

Examples of quantitative indicators that can be collected through CLM		
Area	Indicator	Disaggregation
Care and treatment	Has there been a stock‐out of ARVs in the past month (yes/no)	Type of ARV (name of medicine); regimen (first line, second line third line, paediatric)
Care and treatment	If a stock‐out of ARVs has occurred, how many days did it last before it was resolved?	n/a
Adherence and viral suppression	Number or % of people living with HIV who received their viral load test results within 2 weeks of taking the test (note: does not count if results were returned to clinic—only count when results have been shared with the recipient of care)	Within 2 weeks; within 1 month; more than 1 month

**Examples of qualitative indicators that can be collected through CLM**		
Target audience	Question	Further prompts if needed
Healthcare workers	What are the reasons for stock‐outs of ARVs?	Do communication issues along the supply chain play a role? Does incorrect forecasting and quantification play a role? Do issues with the central medical stores play a role? Does the non‐delivery of orders play a role? Does poor planning play a role? Does reliance on donors play a role? Does non‐payment play a role?
Recipients of care	What are the reasons for people not receiving a viral load test?	Does the knowledge that people living with HIV have of viral load testing guidelines play a role? Do long waiting times at the health facility play a role? Does the availability of working viral load testing machines play a role? Do delays in returning results to recipients of care play a role? Do human resource challenges play a role? Do stock‐outs of lab supplies play a role?

Abbreviations: ARV, Antiretrovirals; CLM, community led monitoring.

### 1.6 Step 3: Engagement

Community implementers analyse the CLM data—recommended in partnership with an advisory group, academic institution or other data expertise—and present the results for discussion among stakeholders who can bring action to bear on the issues identified. ITPC's model recommends the creation of a Community Consultative Group (CCG) (or equivalent), a convening body of 10−15 stakeholders including recipients of care, healthcare providers, health facility managers, donors and government representatives to review the data, harvest insights and co‐create solutions [13].

Beyond the CCG, Operational research indicates that CLM data collectors play a vital but often informal role in reporting CLM data to healthcare providers, sharing trends back to communities and engaging communities in HIV education. In 2023, we consulted 306 CLM stakeholders through surveys (*n* = 188) and interviews (*n* = 118). Close to half (45%) said they prefer to receive CLM feedback in real time, during data collection. Many (61%) said data collectors are present during CLM feedback sessions, often leading discussions. More than half (53%) said they remember CLM data better if a person living with HIV or key population present it to them. Healthcare workers say the “full participation of all people involved” helps them act on CLM data. One directly credits data collectors as catalysts: “It was the data collector who linked us up with the District Health Office when we had a stockout and the issue was sorted immediately.”

### 1.7 Step 4: Advocacy

When stakeholder‐shared issues remain unresolved, CLM escalates to advocacy. CLM‐derived insights directly trigger programme improvements: for instance, qualitative interviews identified healthcare providers’ fear of losing clinical oversight as a barrier to scaling up the multi‐month dispensing of antiretroviral therapy (ART). However, this concern contradicts clinical evidence, as demonstrated by a cluster‐randomized trial in Malawi demonstrating non‐inferiority of 6‐month ART dispensing [20]. Implementing partners in Malawi advocated for incorporating this evidence into clinical trainings, and documented a substantial increase in multi‐month dispensing of ART during COVID‐19 (from 6% to 31%). This case exemplifies CLM's ability to swiftly identify discrepancies between normative guidance and actual practice, leading to rapid improvements in service delivery, such as healthcare provider trainings, outpacing traditional PS approaches.

## 2 DISCUSSION

### 2.1 Evidence of CLM impact

PS approaches that centre community leadership and ownership may provide additional opportunities for impact assessment. In South Africa, our community partners leveraged their good relationship with the West Rand District Health Service to gain full access to the DHIS2 and the Stock Visibility System. This level of access provides a unique opportunity to calculate odds ratios and assess whether the targeted CLM‐derived interventions implemented at monitored sites correlate with improved health outcomes. Interventions include service demand generation, healthcare worker training, feedback with facility managers and engaging clinic committees on identified gaps. Sites with CLM mechanisms in place are more likely to initiate people onto pre‐exposure prophylaxis (PrEP) following an HIV test (1.32 OR 95% CI 1.27−1.38), more likely to find and diagnose adolescent girls and young women living with HIV (1.46 OR 95% CI 1.28−1.66), and more likely to ensure pregnant women deliver in the health facility (1.99 OR 95% CI 1.51−2.62) [21].

CLM not only provides an immediate of reactive path to address problems, but it also offers opportunities to anticipate and mitigate potential issues within communities and programmes before they arise. For instance, during the onset of COVID‐19, community data allowed for the timely identification of challenges stemming from emergency measures, such as difficulties accessing monthly ART due to lockdowns, curfews and social distancing mandates. Communities were able to use CLM to facilitate the swift implementation of remedial measures, such as multi‐month dispensing, to address these challenges.

The CLM approach is not limited to using local data for local change. Community‐derived indicators can also enhance global data frameworks. In September 2020, ITPC and its partners implemented data collection on multi‐month dispensing of ART in response to community concerns about its importance for people living with HIV during COVID‐19. This indicator's specificity and relevance extended beyond the project, leading UNAIDS to include it as a new indicator in the Global AIDS Monitoring framework in February 2022 [22].

### 2.2 Other CLM models

Similar CLM approaches have been successfully applied in diverse settings, including customer satisfaction surveys in Zimbabwe [3], peer‐led facility exit interviews in Papua New Guinea [23], community‐based focus groups and semi‐structured interviews in Haiti [24], “mystery shoppers” in Jamaica and the United States [25, 26], clinic records surveys in Sierra Leone [2] and a mix of these methods in a regional community treatment observatory in West Africa [27].

CLM frameworks should always be context‐dependent and community‐defined. A limited number of CLM initiatives combine both qualitative and quantitative methods, which is recommended for triangulation [11, 28, 29]. Further, while many CLM initiatives have done well to identify problems and barriers to care, few have effectively documented how identifying these problems has led to concrete changes and programme improvements [9].

### 2.3 Limitations of CLM

The limitations of CLM have primarily to do with implementation. A surge in CLM investments by major bilateral and multilateral donors has led to a rush to establish CLM programmes, driven by pressure to produce primarily quantitative data and reach targets. This urgency undermines the crucial preparation steps that are necessary for the creation of effective CLM—such as the establishment of communities firmly in the lead of the process, including determining which issues will be monitored via CLM indicators; briefing of government and health facility partners on the objectives and nature of the monitoring that will take place, as there can often be scepticism when data collectors seek to begin frequenting monitoring sites; and the shared objective of CLM that is oriented towards “fact‐finding” rather than “fault‐finding” with the ultimate goal of service improvement. It is imperative to uphold the fidelity and rigour of the CLM model, as partial CLM or other forms of monitoring or community data collection are not equivalent. CLM implementation must be technically sound to be credible and politically viable. Likewise, multi‐year programmes are best, as these allow for the ebb and flow of the relationships among all partners and enable time for the CCG mechanism to become well‐established and begin producing impactful results—but funding sources often move from year to year with extremely high expectations for producing data first and foremost.

Quantifying the specific impact of CLM can be difficult. Measuring the impact of CLM is complex, as the structure of most CLM programmes does not allow for direct comparison with “control groups” that would isolate the effect of CLM. Further work is needed to triangulate control versus intervention group comparisons, such as through healthcare worker testimonials about how the intervention may have helped, alongside other qualitative and quantitative data sources. Randomized control studies on CLM are needed.

### 2.4 Outstanding questions for further inquiry

Significant questions remain about CLM. How many facilities must be monitored to have sufficient data to identify and initiate programme improvements? When do facilities “graduate” from CLM, and what kind of periodic evaluation is needed to sustain gains over the long term? What barriers hinder CLM data from being perceived as credible by decision‐makers? And what factors determine the optimal blend of analysis, advocacy, diplomacy and accountability needed to transform CLM data from mere statistics into tangible and meaningful improvements in the lives of recipients of care?

## 3 CONCLUSIONS

CLM embodies a shift from data extraction to data democracy, empowering those most affected by health systems to drive evidence‐informed change that leads to health programme improvements. This reframing is indispensable for ensuring that PS effectively identifies, measures, addresses and monitors inequities.

Traditional science methods may incorporate input from care recipients but often lack direct community involvement in programme design and adaptation. CLM offers a remedy by empowering communities to both generate and govern data. Elevating communities from mere data sources to data experts transforms power dynamics inherent in PS and enhances its effectiveness.

Recognizing CLM as an investment in community systems, rather than just data and programmes, is vital for its sustainability. Viewing CLM solely as a means to extract data risks undermining its potential for community leadership in monitoring and accountability. When communities are engaged as leaders and experts, as demonstrated by CLM, programmes become more relevant, evidence‐based and impactful, leading to more sustainable and effective health interventions.

Incorporating CLM into PS ensures interventions are not only relevant and impactful but also aligned with the needs and perspectives of the communities they serve.

## COMPETING INTERESTS

The authors declare that they have no competing interests.

## AUTHORS’ CONTRIBUTIONS

SLB designed the commentary. KJL drafted the manuscript and critically revised it. All other authors (MS, CAK, DM and GO) provided insights and comments. All authors have read and approved the final manuscript.

## Data Availability

The data that support the findings of this study are available on request from the corresponding author. The data are not publicly available due to privacy or ethical restrictions.

## References

[1] Anam FR , Nkosi S , Sebayang M , Jokonya M , Dunaway K , El Alaoui T . Let us lead: community leadership in the AIDS response is its fundamental pillar for success. J Int AIDS Soc. 2023;26(12):e26196. 38031904 10.1002/jia2.26196 PMC10687760

[2] Ellie MP , Kibe PW , Flomo BM , Ngwatu BK . Breaking barriers: using evidence from a Community Treatment Observatory to enhance uptake of HIV services in Sierra Leone. J Health Des. 2019;4(1):163–167.

[3] Makoni T , Madzima B , Dzinamarira T , Moyo E , Musuka G. Putting communities at the forefront of community‐led monitoring in Zimbabwe. Front Public Health. 2024;11:1320944. 38259750 10.3389/fpubh.2023.1320944 PMC10801175

[4] ITPC . “They keep us on our toes”: how the Regional Community Treatment Observatory in West Africa improved HIV service delivery, strengthened systems for health, and institutionalized community‐led monitoring. Johannesburg, South Africa: ITPC; 2020.

[5] Tshuma N , Elakpa DN , Moyo C , Soboyisi M , Moyo S , Mpofu S , et al. The transformative impact of community‐led monitoring in the South African health system: a comprehensive analysis. Int J Public Health. 2024;69:1606591. 38420516 10.3389/ijph.2024.1606591 PMC10899425

[6] PEPFAR . PEPFAR 2023 Country and Regional Operational Plan (COP/ROP) Guidance for all PEPFAR‐Supported Countries. 2023. Accessed April 11, 2024. Available from: https://www.state.gov/wp‐content/uploads/2023/07/PEPFAR‐2023‐Country‐and‐Regional‐Operational‐Plan.pdf

[7] Global Fund . Technical Review Panel Observations from the 2020–2022 Allocation Period Summary of Recommendations. Geneva, Switzerland; 2023.

[8] Global Fund . Community‐based monitoring: an overview. Geneva, Switzerland; 2020.

[9] UNAIDS . Community‐led monitoring in action: emerging evidence and good practice. Geneva, Switzerland; 2023.

[10] Becker M , Mishra S , Aral S , Bhattacharjee P , Lorway R , Green K , et al. The contributions and future direction of Program Science in HIV/STI prevention. Emerg Themes Epidemiol. 2018;15(1):1–7. 29872450 10.1186/s12982-018-0076-8 PMC5972407

[11] Joint United Nations Program on HIV/AIDS . Establishing community‐led monitoring of HIV services. Geneva, Switzerland; 2021.

[12] Baum F , MacDougall C , Smith D . Participatory action research. J Epidemiol Community Health. 2006;60:854–857. 16973531 10.1136/jech.2004.028662 PMC2566051

[13] Jallow W , Swan T , Oberth G , Baptiste S , Rafif N , Moreno CGdL , et al. How to implement community‐led monitoring: a community toolkit. ITPC; 2021.

[14] Baptiste S , Manouan A , Garcia P , Etya'ale H , Swan T , Jallow W . Community‐led monitoring: when community data drives implementation strategies. Curr HIV/AIDS Rep. 2020;17:415–421. 32734363 10.1007/s11904-020-00521-2 PMC7497354

[15] Björkman M , Svensson J . Power to the people: evidence from a randomized field experiment on community‐based monitoring in Uganda. Q J Econ. 2009;124(2):735–769.

[16] Kakade D . Community‐based monitoring as an accountability tool: influence on rural health services in Maharashtra, India. BMC Proc. 2012;6(Suppl 1):O9.

[17] ITPC . Data for a difference: key findings, analysis and advocacy opportunities from the regional Community Treatment Observatory in West Africa. Johannesburg, South Africa: International Treatment Preparedness Coalition; 2019.

[18] EpiC . Community‐led monitoring technical guide. Durham NC: FHI 360; 2021.

[19] Georgetown University Law Center O'Neill Institute for National and Global Health Law . Community‐led monitoring: best practices for strengthening the model. Washington, DC: Georgetown University Law Center; 2022.

[20] Hoffman RM , Moyo C , Balakasi KT , Siwale Z , Hubbard J , Bardon A , et al. Multimonth dispensing of up to 6 months of antiretroviral therapy in Malawi and Zambia (INTERVAL): a cluster‐randomised, non‐blinded, noninferiority trial. Lancet Glob Health. 2021;9(5):e628–e638. 33865471 10.1016/S2214-109X(21)00039-5

[21] ITPC . Insight, influence, and impact: 10 big change stories from the Citizen Science Community‐led Monitoring Project in 2023. 2024.

[22] UNAIDS . Global AIDS Monitoring 2022: indicators and questions for monitoring progress on the 2021 Political Declaration on HIV and AIDS. 2022. p. 106. Accessed January 16, 2024. Available from: https://www.unaids.org/sites/default/files/media_asset/UNAIDS_GAM_Framework_2022_EN.pdf

[23] Hakim A . HIV prevalence and healthcare service utilization among female sex workers, men who have sex with men, and transgender women in Papua New Guinea [dissertation]. Ghent University; 2022.

[24] Policar S , Sharp A , Isidor Hyppolite J , Alfred GM , Steide E , Lucien L , et al. Drivers of HIV treatment interruption: early findings from community‐led monitoring program in Haiti. PLoS One. 2023;18(12):e0295023. 38051706 10.1371/journal.pone.0295023 PMC10697516

[25] Ministry of Health and Wellness . Global AIDS Monitoring Report Jamaica 2020. 2020. Accessed March 25, 2024. Available from: https://www.unaids.org/sites/default/files/country/documents/JAM_2020_countryreport.pdf

[26] Bauermeister JA , Pingel ES , Jadwin‐Cakmak L , Meanley S , Alapati D , Moore M , et al. The use of mystery shopping for quality assurance evaluations of HIV/STI testing sites offering services to young gay and bisexual men. AIDS Behav. 2015;19:1919–1927. 26303197 10.1007/s10461-015-1174-z PMC4567391

[27] Oberth G , Baptiste S , Jallow W , Manouan A , Garcia P , Traore AM , et al. Understanding gaps in the HIV treatment cascade in eleven West African countries: findings from a regional Community Treatment Observatory. Centre for Social Science Research (CSSR); 2019.

[28] ITPC . Community‐led monitoring: guide to support CLM data use in decision‐making. Johannesburg, South Africa; 2023.

[29] International AIDS Society (IAS) . Community‐led monitoring of programs and policies related to HIV, tuberculosis, and malaria: a guide to support inclusion of CLM in funding requests to the Global Fund. 2022. Accessed November 8, 2023. Available from: https://cquin.icap.columbia.edu/wp‐content/uploads/2022/12/IAS‐CLM‐Guide‐final.pdf

